# How Epstein-Barr Virus Induces the Reorganization of Cellular Chromatin

**DOI:** 10.1128/mbio.02686-22

**Published:** 2023-01-10

**Authors:** Quincy Rosemarie, Elijah Kirschstein, Bill Sugden

**Affiliations:** a Department of Oncology, McArdle Laboratory for Cancer Research, School of Medicine and Public Health, University of Wisconsin—Madison, Madison, Wisconsin, USA; Princeton University

**Keywords:** Epstein-Barr virus, chromatin reorganization, productive infection, virus-host interactions

## Abstract

We have discovered how Epstein-Barr virus (EBV) induces the reorganization of cellular chromatin (ROCC), in which host chromatin is compacted and marginated within the nucleus, with viral DNA replication occurring in the chromatin-free regions. Five families of DNA viruses induce ROCC: herpesviruses, adenoviruses, parvoviruses, baculoviruses, and geminiviruses. These families infect a variety of hosts, including vertebrates, insects, and plants. They also share several characteristics: they replicate and encapsidate their genomes in the host nucleus and package their genomes unbound by histones. We have identified the viral genes and processes required for EBV’s ROCC. Each of EBV’s seven core DNA synthesis genes and its origin of lytic replication (*oriLyt*), in *trans*, are required, while its protein kinase, BGLF4, and its true late genes are not. Following these findings, we tested the role of EBV lytic DNA amplification in driving ROCC. Surprisingly, the inhibition of EBV’s lytic DNA synthesis still supports chromatin compaction but blocks its margination. We propose a two-step model for ROCC. First, the initiation of viral lytic DNA synthesis induces a cellular response that results in global chromatin compaction. Second, the histone-free, productive viral DNA synthesis leads to the margination of compacted chromatin to the nuclear periphery. We have tested this model by asking if the histone-associated simian virus 40 (SV40) DNA synthesis could substitute for *oriLyt*-mediated synthesis and found that EBV’s ROCC is incompatible with SV40 DNA replication. Elucidating EBV’s induction of ROCC both illuminates how other viruses can do so and indicates how this spatial control of cellular chromatin benefits them.

## INTRODUCTION

Viruses as obligate cellular parasites evolve to manipulate cellular functions for their own benefit. An example of one such manipulation is illustrated by Epstein-Barr virus (EBV) and found in members of four additional families of viruses which expropriate control of nuclear structure during their productive phases. These viruses foster the compaction and extensive nuclear margination of their host cell’s DNA, constituting an extensive reorganization of cellular chromatin (ROCC).

Viral ROCCs are characterized in part by the spatial compaction of cellular chromatin. DNA compaction is a fundamental mechanism used by organisms, cells, and viruses to organize their genetic material. It contributes to equal partitioning of genomic material during cell division, control of gene expression, and the packaging of DNAs into small volumes. Cells compact their chromosomal DNA to ensure equal partitioning to daughter cells during mitosis. This compaction occurs through the coiling of histone-associated DNA (chromatin) into increasingly higher-order structures, resulting in compacted chromosomes (reviewed in references 1 and 2). In a typical uninfected cell, metaphase chromosomes are the most extreme form of DNA compaction. Compaction of DNA also can control gene expression. Cellular chromatin exists as heterochromatin, in which it is histone rich and condensed, or as euchromatin, in which it is lightly packed, with fewer histones (reviewed in references 3 and 4). Heterochromatin is less accessible to transcriptional machineries and is typically associated with diminished gene expression. In contrast, the “open” euchromatin is frequently associated with regions of active gene expression.

Less generally, compaction allows DNA to be packaged into small volumes. One extreme example occurs in sperm, in which their haploid genome is compacted by >50-fold (5, 6). Viruses, too, tightly pack their genomes within viral particles. EBV, a herpesvirus, compacts its genome >50-fold in its virus particles relative to the human genome in a cell nucleus (see Text S1 in the supplemental material). These compactions are in part achieved by the absence of histones from packaged DNA. Only 15% of sperm DNA is associated with histones; in the other 85%, histones are replaced by protamines (6). Similarly, the EBV genome, as well as those of several other DNA viruses, while chromatinized within its host cell, is not associated with cellular histones in its virus particles. Some viruses encode histone-like proteins that associate with their genomes within virus particles; for example, baculoviruses encode P6.9 (7–9) and adenoviruses encode protein VII (10). However, it appears that, at least in adenoviruses, these histone-like proteins are not required for their genome packaging (10).

10.1128/mbio.02686-22.1 TEXT S1 Calculation of DNA compaction within EBV virion. Download Text S1, DOCX file, 0.01 MB. Copyright © 2023 Rosemarie et al. 2023 Rosemarie et al. https://creativecommons.org/licenses/by/4.0/ This content is distributed under the terms of the Creative Commons Attribution 4.0 International license.

We have elucidated a second way in which viruses dramatically compact DNA, this time, their host cell’s chromatin. Five families of viruses induce the reorganization of cellular chromatin (ROCC) during their productive phases: herpesviruses (11–13), adenoviruses (14, 15), parvoviruses (16), baculoviruses (17), and geminiviruses (18–20). Notably, these virus families span a variety of hosts, including vertebrates, insects, and plants. These viruses’ ROCC involves the spatial compaction and margination of host chromatin toward the periphery of the nucleus, away from viral DNA replication factories. Given that ROCC occurs in five families of viruses, it is likely integral to their life cycles. We have examined ROCC as induced by EBV, both genetically and mechanistically, to elucidate this form of DNA compaction.

EBV, a gammaherpesvirus, induces ROCC. It has two phases in its life cycle: latent and lytic (reviewed in reference 21). EBV’s latent phase occurs when the virus infects and then is maintained in host cells, with few viral genes expressed and no production of viral progeny. On rare occasions, the latent virus reactivates to enter the lytic phase, in which high levels of viral gene expression occur and progeny virus particles are produced. During this productive, lytic phase, EBV compacts and marginates its host’s chromatin, thus inducing ROCC (13).

We have first established the requirements for EBV to induce ROCC. (i) Using a single-gene knockout (KO) library of EBV mutants, we have found that each of EBV’s seven core lytic DNA synthesis genes and its origin of lytic replication (*oriLyt*) are required for ROCC. (ii) Extending this test to additional candidate genes revealed that EBV’s protein kinase, BGLF4, and the viral complement of true late genes are not required for ROCC. (iii) We further investigated the role of EBV’s DNA synthesis for ROCC and found that the enzymatic function of the viral DNA polymerase, BALF5, is essential for ROCC. (iv) However, when we specifically blocked EBV’s lytic DNA synthesis using two inhibitors, ganciclovir (GCV) and phosphonoacetic acid (PAA), we unexpectedly found that detectable lytic DNA synthesis is not required for chromatin compaction but is required for its margination. Thus, we propose a model in which ROCC requires two steps: first, initiation of viral DNA synthesis induces a cellular response that results in global chromatin compaction; then, extensive accumulation of histone-free, newly synthesized viral DNA leads to the margination of the compacted cellular chromatin to the periphery of the nucleus.

In order to understand ROCC further, we have explored it comparatively. We examined EBV’s ROCC in cells cotransfected with simian virus 40 (SV40), a DNA virus that packages chromatinized DNA and does not support ROCC (22, 23). We found that cells in which the first step of ROCC has been triggered do not support SV40 DNA amplification. Thus, EBV’s ROCC is incompatible with SV40’s histone-associated DNA synthesis.

## RESULTS

### EBV’s *oriLyt* and DNA synthesis genes are required for ROCC.

**FIG 1:**
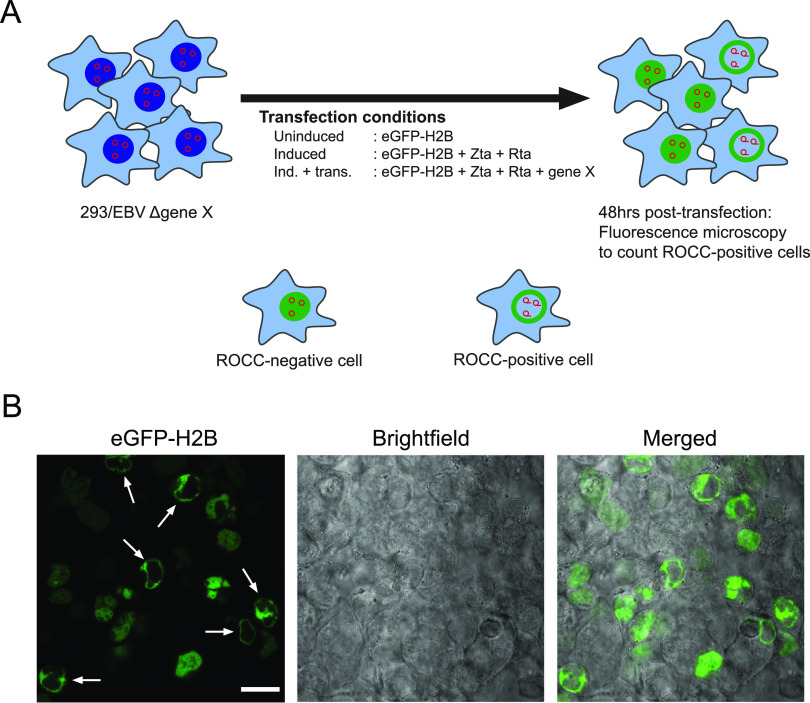
Transcomplementation assay of single-gene EBV KO mutants to assess the requirement of the KO genes for ROCC. (A) Experimental workflow of ROCC transcomplementation assay. 293 cell lines stably maintaining EBV BACmids with a single-gene knockout were transfected under three different conditions: uninduced, induced, and induced and transcomplemented (Ind. + trans., also called I+t). Cells under all three conditions were transfected with an eGFP-H2B (or mCherry-H2B) expression vector to allow visualization of host chromatin. At 48 h posttransfection, cells were visualized with live-cell fluorescence microscopy and the fraction of cells displaying ROCC was measured. (B) Representative image of 293/EBV ΔBALF5 cells induced and transcomplemented (I+t), taken using scanning confocal microscopy. White arrows indicate ROCC^+^ cells. Bar, 20 μm.

EBV’s ROCC occurs in parallel with lytic DNA synthesis; therefore, we investigated any requirement of EBV’s DNA synthesis genes for ROCC. The origin of lytic replication (*oriLyt*) and seven early genes (*BALF5*, *BALF2*, *BBLF2/3*, *BBLF4*, *BSLF1*, *BMLF1*, and *BMRF1*) were found to be necessary and, in combination with *BZLF1* and *BRLF1*, sufficient to support this synthesis (24, 25) (Table 1 contains a list of genes and their functions). Cells from either of two libraries of EBV (26, 27), in which mutants null for a single EBV gene were maintained in 293 cells, were used for transcomplementation assays (Fig. 1A). Each cell line was transfected with an expression vector for enhanced green fluorescent protein (eGFP)-H2B or mCherry-H2B to visualize cellular chromatin and with an empty expression vector (uninduced [U]), expression vectors for EBV’s BZLF1 and BRLF1 (induced [I]), or expression vectors for BZLF1, BRLF1, and the missing gene (induced + transcomplemented [I+t]). At 48 h posttransfection, the cells were viewed by fluorescence microscopy, and the number of cells supporting ROCC was counted (Fig. 1B; see also Fig. S1 in the supplemental material). The proportion of cells in the population that supported ROCC was normalized to that of the transcomplemented (I+t) condition (Table 1; see also Table S1). In all eight sets of assays, uninduced cells did not support ROCC. Samples in the induced group had zero or near-zero cells supporting ROCC, and 2 to 14% of cells in the transcomplemented group supported ROCC. These various levels reflect clonal differences in the rates of entry into the lytic phase. ROCC did not occur in the absence of any of these eight viral genes. Thus, *oriLyt* and each of the seven core lytic DNA synthesis genes are required for ROCC.

**TABLE 1:** Results of measuring ROCC with transcomplementation assays performed on cells that stably maintain mutant EBV genomes with a single gene knocked out^*a*^

KO gene	Gene function	Relative fraction of ROCC^+^ cells^*b*^		
		I+t	I	U
Early genes, core components of lytic DNA synthesis complex				
BALF5	DNA polymerase	1	0*	0
BALF2	ssDNA-binding protein	1	0.05*	0
BBLF2/3	Primase-associated factor	1	0*	0
BBLF4	Helicase	1	0*	0
BSLF1	Primase	1	0.02*	0
BMLF1	RNA processing and export	1	0*	0
BMRF1	Polymerase processivity factor	1	0.03*	0

*oriLyt*	Origin of lytic replication	1	0*	0

Early gene: BGLF4	Serine/threonine kinase	1	0.81	0
Late gene transcription complex				
BVLF1	vPIC component	1	1.96**	0
BcRF1	vPIC component	1	2.68*	0

a I+t, induced + transcomplemented; I, induced; U, uninduced; ssDNA, single-stranded DNA.

b More than 400 eGFP- or mCherry-positive cells were counted per condition (see Table S1), from a combination of three transfection replicates. *, *P* value < 0.01; **, *P* value < 0.05, Fisher’s exact test of I versus I+t group pairs.

10.1128/mbio.02686-22.2 FIG S1 Representative images of induced and induced + transcomplemented EBV-positive cells from the transcomplementation assay (Fig. 1). 293/EBV ΔBSLF1 cells were transfected with expression vectors of eGFP-H2B, Rta, and Zta. Induced + transcomplemented cells were additionally transfected with an expression vector of EBV’s BSLF1. At 48 h posttransfection, cells were fixed, stained with DAPI, and imaged using scanning confocal microscopy. Representative image fields of cells are shown. White arrows indicate ROCC^+^ cells. Bar, 20 μm. Download FIG S1, EPS file, 2.7 MB. Copyright © 2023 Rosemarie et al. 2023 Rosemarie et al. https://creativecommons.org/licenses/by/4.0/ This content is distributed under the terms of the Creative Commons Attribution 4.0 International license.

10.1128/mbio.02686-22.9 TABLE S1 Detailed results of measuring ROCC with transcomplementation assays performed on cells that stably maintain mutant EBV genomes with a single gene knocked out. Download Table S1, DOCX file, 0.02 MB. Copyright © 2023 Rosemarie et al. 2023 Rosemarie et al. https://creativecommons.org/licenses/by/4.0/ This content is distributed under the terms of the Creative Commons Attribution 4.0 International license.

### EBV’s protein kinase, BGLF4, is not required for ROCC.

EBV’s serine/threonine (S/T) kinase, also known as BGLF4 or EBV-PK, has previously been reported to support a phenotype of premature chromosome condensation in transfected cells (28, 29). Given that ROCC involves the spatial condensation of cellular chromatin, we asked if BGLF4 contributes to ROCC, using the same transcomplementation assay as described in Fig. 1. In contrast to EBV BACmids (shuttle vectors for amplifying EBV plasmids in *E. coli*) deleted for *oriLyt* or for any of the seven core lytic DNA synthesis genes, EBV was capable of supporting ROCC in the absence of BGLF4 (Table 1, induced versus induced + transcomplemented proportions of ROCC^+^ cells are not significantly different). These cells were null for BGLF4, as confirmed by BMRF1, a substrate of BGLF4, not being phosphorylated (Fig. S2). This result indicates that, in 293/EBV cells, BGLF4 is not required for ROCC.

10.1128/mbio.02686-22.3 FIG S2 Verification of BGLF4 knockout in 293/EBV ΔBGLF4. 293/EBV ΔBGLF4 cells were transfected under three conditions: uninduced (U; eGFP-H2B), induced (I; eGFP-H2B + Zta + Rta), or induced + transcomplemented (I+t; eGFP-H2B + Zta + Rta + BGLF4). Samples were immunoblotted for the early lytic protein BMRF1, a phosphorylation target of the BGLF4 kinase. I+t cells have both hyper- and hypomethylated BMRF1, whereas induced samples show only hypomethylated BMRF1, confirming that BGFL4 is knocked out in this cell line. As controls, 293FT cells were transfected with expression vectors of BMRF1 with and without BGLF4. β-Actin was immunoblotted as a loading control. Exposure times: 21 s (BMRF1) and 101 s (β-actin). Download FIG S2, EPS file, 1.5 MB. Copyright © 2023 Rosemarie et al. 2023 Rosemarie et al. https://creativecommons.org/licenses/by/4.0/ This content is distributed under the terms of the Creative Commons Attribution 4.0 International license.

### EBV’s true late genes are not required for ROCC.

An additional insight can be gleaned from the Δ*oriLyt* transcomplementation assay. The expression of EBV’s true late gene requires an *oriLyt* sequence in *cis* (on the same DNA), as well as *oriLyt*-mediated DNA replication of the EBV genome containing these genes (27). A true late gene is defined as one that absolutely requires lytic DNA synthesis for its expression, whereas a leaky late gene is expressed at low levels prior to lytic DNA synthesis, with an increase in its expression upon lytic DNA synthesis (30). In the induced + transcomplemented group, the 293/EBV Δ*oriLyt* cell line was transfected with a plasmid encoding *oriLyt*. Under this condition, *oriLyt* is present in *trans* to the late genes, and Djavadian et al. have shown that this configuration does not support the expression of true late genes (27). Despite the lack of late gene expression, Δ*oriLyt* induced + transcomplemented cells support ROCC (Table 1; Table S1). Thus, EBV’s true late genes are not required for ROCC.

The role of true late genes for ROCC was tested independently using cells defective for the expression of true late genes. ΔBVLF1 and ΔBcRF1 cell lines have been previously characterized to be incapable of expressing true late genes (27). BVLF1 and BcRF1 are two of six genes unique to beta- and gammaherpesviruses that form a viral preinitiation complex (vPIC) responsible for true late gene transcription (27, 31). EBV with defective vPICs lacks expression of true late genes (27, 30). Transcomplementation assays of ΔBVLF1 and ΔBcRF1 showed that these cells can support ROCC in the absence of BVLF1 and BcRF1 (Table 1; Table S1). Higher proportions of cells supporting ROCC were found in the induced (I) than in the induced + transcomplementation (I+t) group of these cells. One likely explanation for this observation is that the I+t group was capable of expressing true late genes, following which virus particles were assembled and released, resulting in cell death. At 48 h postinduction (hpi), some cells would have completed their lytic phase and died (13, 32). On the other hand, lytic cells in the induced group were incapable of expressing true late genes, as they were missing a vPIC component, and therefore could survive longer in the lytic phase, leading to their being observed upon cell counting at 48 hpi and adding to the apparent proportion of ROCC-positive cells. Collectively, our findings indicate that EBV’s true late genes are not required for ROCC.

### The enzymatic function of EBV’s DNA polymerase is required for ROCC.

**FIG 2:**
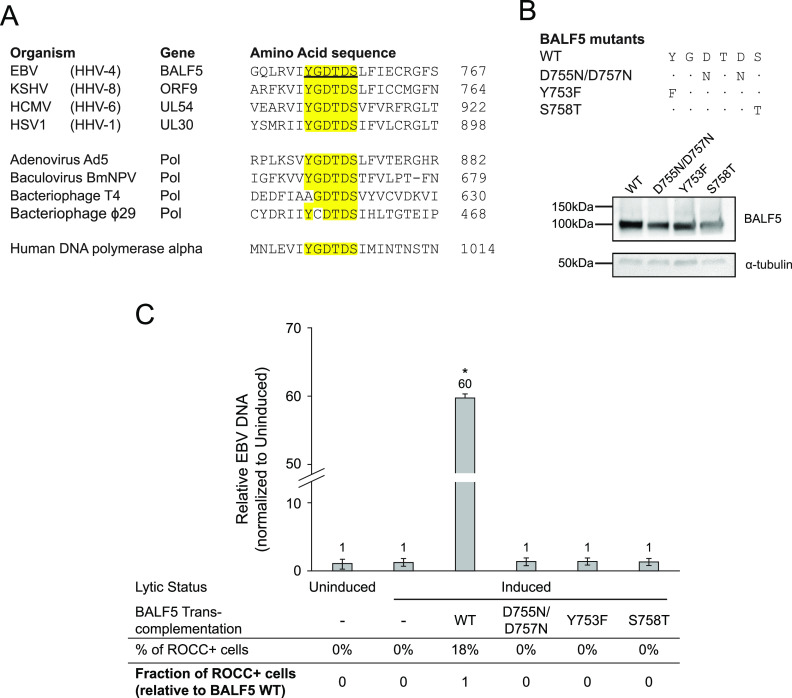
Catalytically dead mutants of EBV’s DNA polymerase BALF5 are incapable of supporting ROCC. (A) Multiple sequence alignment of the well-conserved YGDTDS sequence within region I of DNA polymerases from several different virus families, along with that of human DNA polymerase alpha. Alignment was performed using Clustal O v1.2.4. (B) Schematic of mutant BALF5 variants and their expression level as analyzed by immunoblotting. α-Tubulin is immunoblotted as a loading control. Exposure time: 21 s for BALF5, 201 s for α-tubulin. WT, wild type. (C) 293/EBV ΔBALF5 cell transcomplementation assay with BALF5 variants. Bar plot displaying relative EBV genome measurement by qPCR, normalized to that under the uninduced condition. The corresponding fraction of ROCC^+^ cells is noted below, normalized to induced + transcomplementation with WT BALF5. All three BALF5 mutants are null for its DNA polymerase activity and are incapable of supporting ROCC. Error bars show standard deviations. More than 600 eGFP-positive cells were examined for ROCC per condition, from a combination of three transfection replicates. qPCR measurements were performed on three technical replicates per condition per transfection replicate, from a total of three transfection replicates. *, *P* value of <0.05, Welch’s *t* test of each condition compared to the induced (without transcomplementation) condition.

Given that EBV’s true late genes as well as one of the early genes, BGLF4, are dispensable for ROCC, we further scrutinized the role of EBV’s lytic DNA synthesis for ROCC using mutants of EBV’s DNA polymerase, BALF5. All herpesvirus DNA polymerases belong to the alpha-like polymerase family, which has seven highly conserved regions (33–35). The most conserved region, region I, is centered around the amino acid residue sequence YGDTDS, which is invariant in herpesvirus polymerases, those of many other viruses, and the human DNA polymerase alpha (Fig. 2A) (34, 36, 37). Region I is located within the palm domain of the DNA polymerase (Fig. S3) and is involved in the coordination of metal ions at the catalytic site (38, 39). Three BALF5 mutants were generated, with substitutions in the YGDTDS residues: D755N/D757N, Y753F, and S758T. The two aspartic acid residues (D755 and D757 in EBV’s BALF5) appear to be particularly sensitive to mutations, as their substitutions typically lead to functionally null polymerases, as has been shown in both human DNA polymerase alpha and herpes simplex virus 1 (HSV-1) DNA polymerase (Pol) (37, 40).

10.1128/mbio.02686-22.4 FIG S3 Crystal structure of the HSV1 DNA polymerase, a homologue to EBV’s BALF5, showing the highly conserved YGDTDS sequence. (Left) The HSV-1 DNA polymerase X-ray crystal structure has been published (S. Liu, J. D. Knafels, J. S. Chang, G. A. Waszak, et al., J Biol Chem 281:18193–18200, 2006, https://doi.org/10.1074/jbc.M602414200) and is publicly available (PDB code 2GV9). (Right) The highly conserved YGDTDS sequence in region I, located within the palm domain, is shown at higher magnification. Protein structures were visualized using PyMOL v2.4. We were unable to find a full-length EBV BALF5 crystal structure which had been published at the time of the manuscript’s publication. Download FIG S3, EPS file, 2.1 MB. Copyright © 2023 Rosemarie et al. 2023 Rosemarie et al. https://creativecommons.org/licenses/by/4.0/ This content is distributed under the terms of the Creative Commons Attribution 4.0 International license.

We constructed FLAG-tagged expression vectors for the D755N/D757N, Y753F, and S758T BALF5 mutants and used them in transcomplementation assays in the 293/EBV ΔBALF5 cell line. Control transcomplementation experiments using FLAG-tagged wild-type BALF5 showed that the tagged protein was functional in supporting lytic DNA synthesis and ROCC. Western blotting assays and quantitative PCR (qPCR) measurements verified the expression of these mutant constructs and the effects of these mutations on BALF5’s activity (Fig. 2B and C; see also Table S2). All three mutants were null for BALF5 activity. These BALF5 mutants failed to support ROCC (Fig. 2C). This finding indicates that the complete absence of lytic EBV DNA synthesis leads to a complete abrogation of ROCC.

10.1128/mbio.02686-22.10 TABLE S2 Results of measuring ROCC with 293/EBV ΔBALF5 transcomplementation assay. Download Table S2, DOCX file, 0.01 MB. Copyright © 2023 Rosemarie et al. 2023 Rosemarie et al. https://creativecommons.org/licenses/by/4.0/ This content is distributed under the terms of the Creative Commons Attribution 4.0 International license.

### Detectable EBV lytic DNA synthesis is not required for some features of ROCC.

**FIG 3:**
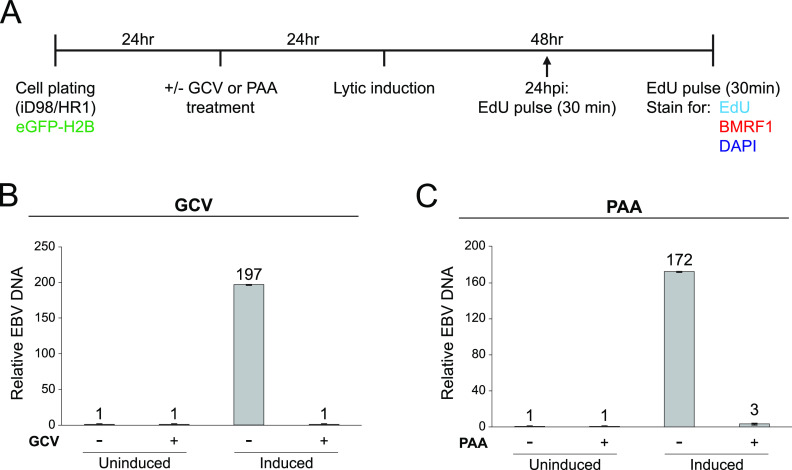
GCV and PAA treatment inhibits EBV lytic DNA synthesis. (A) Experimental workflow for ROCC assay with drug-mediated inhibition of viral DNA synthesis. iD98/HR1 cells were treated with ganciclovir (GCV; 40 μg/mL) or phosphonoacetic acid (PAA; 100 μg/mL) 24 h prior to lytic induction. Cells were induced to enter the lytic phase with 200 nM 4-hydroxytamoxifen (4-OHT). At 24 and 48 h postinduction (hpi), cells were pulsed with 10 μM EdU for 30 min. Samples were fixed and subjected to click chemistry for detection of EdU, immunofluorescence for the early protein BMRF1, and DAPI staining for DNA. Imaging was performed using scanning confocal microscopy. (B and C) qPCR measurements of EBV genome from GCV and PAA experiments, normalized to the uninduced, untreated condition. qPCR measurements were performed on three technical replicates per condition per transfection replicate, from a total of three transfection replicates. Error bars show standard deviation.

**FIG 4:**
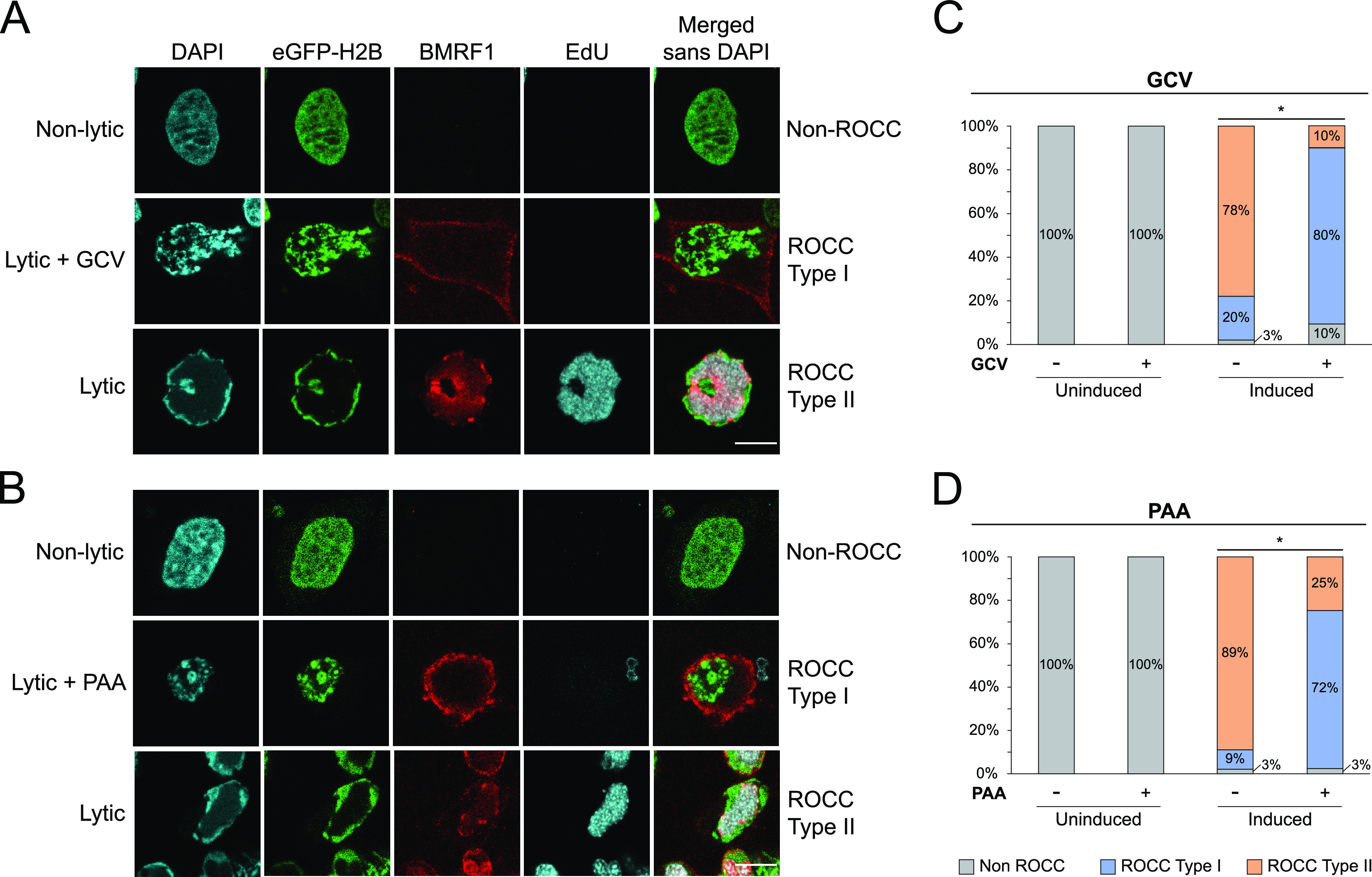
Detectable lytic DNA synthesis is dispensable for chromatin condensation but required for its margination. iD98/HR1 cells were treated with GCV or PAA and induced to enter the lytic phase (Fig. 3A). (A and B) Representative cells from GCV (A) and PAA (B) experiments are shown. Uninduced (nonlytic) cells do not express BMRF1 and do not display ROCC. Induced, lytic cells robustly express BMRF1, have EdU signals localizing to viral replication compartment(s), and support ROCC type II, in which cellular chromatin is both condensed and marginated. Lytic cells treated with GCV or PAA robustly express BMRF1, and ≥90% lack EdU signals, confirming the inhibition of viral DNA synthesis in them. The majority of these cells display ROCC type I, in which cellular chromatin is condensed but not marginated. All images have the same scale; scale bar is 10 μm. (C and D) ROCC types were classified for each imaged cell (all cells in uninduced samples and BMRF1^+^ cells in induced samples) and quantified for each treatment group. Induced cells treated with GCV or PAA support increased levels of ROCC type I compared to induced, untreated cells, which support mostly ROCC type II. *n* > 30 cells per group. *, *P* value of <0.01, 2 × 3 Fisher’s exact test.

The observed requirement for ROCC for all EBV genes essential for viral DNA amplification made it likely that blocking DNA synthesis would block ROCC, too. We tested this hypothesis and uncovered a surprising result. We treated the lytic-phase-inducible, EBV-positive cell line iD98/HR1 (13) with ganciclovir (GCV; 40 μg/mL) or phosphonoacetic acid (PAA; 100 μg/mL) to inhibit viral DNA synthesis. GCV is a guanosine analogue that specifically inhibits the elongation of viral DNA (41, 42). PAA is a pyrophosphate analogue that binds directly to the viral DNA polymerase, thus specifically inhibiting the viral DNA synthesis (43, 44). The iD98/HR1 cells were induced for the lytic phase 24 h following treatment with GCV or PAA and pulsed with 5-ethynyl-2′-deoxyuridine (EdU) for 30 min at 24 and 48 h following induction (Fig. 3A). At 48 hpi, the cells were either harvested for qPCR or fixed for immunofluorescence assays. The viability of cells at 48 hpi was also assessed, and cells were found to be viable (Fig. S4). qPCR measurement for EBV genomes confirmed that GCV or PAA treatment inhibited lytic DNA synthesis to levels comparable to that of uninduced, nonlytic cells (Fig. 3B and C). For immunofluorescence, fixed cells were permeabilized, following which click chemistry was performed to detect EdU signals, and the cells were subjected to immunofluorescence to detect the early lytic protein, BMRF1, and 4′,6-diamidino-2-phenylindole (DAPI) staining to detect DNA. The distribution of BMRF1 differs in cells treated with GCV or PAA and covers the periphery of the nucleus, as has been observed in similarly treated human cytomegalovirus (HCMV)-infected cells (12). Cells that did not express BMRF1 were considered nonlytic, did not accumulate EdU in viral amplification factories, and did not display ROCC (Fig. 4A and B). Only cells found to express BMRF1 were considered to be in the lytic phase. As expected, these cells did have EdU signals in viral amplification factories and did display ROCC. The GCV- or PAA-treated lytic cells, also characterized by BMRF1 expression, largely had no detectable EdU signals (10% or 0% EdU^+^ cells in GCV- or PAA-treated cells, respectively), confirming the inhibition of viral DNA synthesis in them. Two types of ROCC were identified: ROCC type I, in which the cellular chromatin was spatially condensed but not marginated, and ROCC type II, in which the cellular chromatin was spatially condensed and marginated, as seen in wild-type viral productive phase (Fig. 4A and B; see also Fig. S5). These findings were also observed in a different EBV-positive cell line, the gastric carcinoma cell line AGS-Akata (Fig. S6). Quantification of the ROCC types in iD98/HR1 showed that, in those cells treated with GCV or PAA that entered the lytic phase as indicated by their expression of BMRF1, there was a significant increase in ROCC type I (Fig. 4C and D). This finding indicates that detectable EBV lytic DNA synthesis is not required for cellular chromatin condensation but contributes to its margination. Along with findings from the transcomplementation assays with BALF5 mutants, this observation gives us a mechanistic insight into ROCC: the initiation of EBV DNA synthesis is required for ROCC type I. It is yet unknown what other factors are required to support ROCC type I or whether the initiation of EBV’s lytic DNA synthesis is sufficient. Our results indicate that, following the global chromatin compaction characterizing ROCC type I, extensive amplification of EBV genomes gives rise to chromatin margination (ROCC type II).

10.1128/mbio.02686-22.5 FIG S4 ROCC phenotypes are not a property of dead cells. iD98/HR1 eGFP-H2B cells were untreated or pretreated with 100 μg/mL PAA and induced to enter the lytic phase (Fig. 4). Cell viability was assessed by DAPI dye exclusion. (A) Populations of cells that were uninduced, induced, or induced + PAA were observed using live-cell fluorescence microscopy, and the number of DAPI^+^ cells was counted. The proportions of DAPI^+^ cells between all three sample groups were 4% or less. *n* > 100 cells per group. (B) Cells supporting ROCC type I (in the induced + PAA sample) and those supporting ROCC type II (in the induced sample) were observed using eGFP-H2B signals, and the number of DAPI^+^ cells among them was counted. The proportions of ROCC-supporting cells that are DAPI^+^ (nonviable cells) were 3% or less, and these proportions are similar to those at the population level of all three sample groups (uninduced, induced, and induced + PAA). Thus, ROCC phenotypes are not a property of dead cells. *n* > 30 cells per group. Download FIG S4, EPS file, 0.8 MB. Copyright © 2023 Rosemarie et al. 2023 Rosemarie et al. https://creativecommons.org/licenses/by/4.0/ This content is distributed under the terms of the Creative Commons Attribution 4.0 International license.

10.1128/mbio.02686-22.6 FIG S5 Representative images of cells not displaying ROCC or displaying ROCC type I or type II. iD98/HR1 cells stably maintaining an expression vector for eGFP-H2B were imaged using scanning confocal microscopy. Various types of ROCC were captured, and representative images are shown. Non-ROCC cells have their cellular chromatin distributed largely evenly across the nucleus. Cells with ROCC type I have their chromatin condensed but not marginated, whereas those with ROCC type II have their chromatin condensed and marginated toward the periphery of the nucleus. All images have the same scale; bar, 10 μm. Download FIG S5, EPS file, 2.6 MB. Copyright © 2023 Rosemarie et al. 2023 Rosemarie et al. https://creativecommons.org/licenses/by/4.0/ This content is distributed under the terms of the Creative Commons Attribution 4.0 International license.

10.1128/mbio.02686-22.7 FIG S6 EBV’s induction of ROCC and the effects of GCV and PAA on ROCC types are found in multiple distinct EBV-positive cell lines. ROCC was also examined in AGS-Akata cells, which are gastric carcinoma cells superinfected with EBV. Cells induced into EBV’s lytic phase robustly express BMRF1 (red) and support ROCC type II, as assessed by DAPI staining of DNA (cyan) (top panel). Cells induced into EBV’s lytic phase in the presence of 40 μg/mL GCV or 100 μg/mL PAA also robustly express BMRF1 (red) but generally support ROCC type I (cyan, DAPI staining) (middle and bottom panels). All images have the same scale; bar, 10 μm. Download FIG S6, EPS file, 2.5 MB. Copyright © 2023 Rosemarie et al. 2023 Rosemarie et al. https://creativecommons.org/licenses/by/4.0/ This content is distributed under the terms of the Creative Commons Attribution 4.0 International license.

### EBV’s ROCC is incompatible with the DNA replication of the non-ROCC SV40.

**FIG 5:**
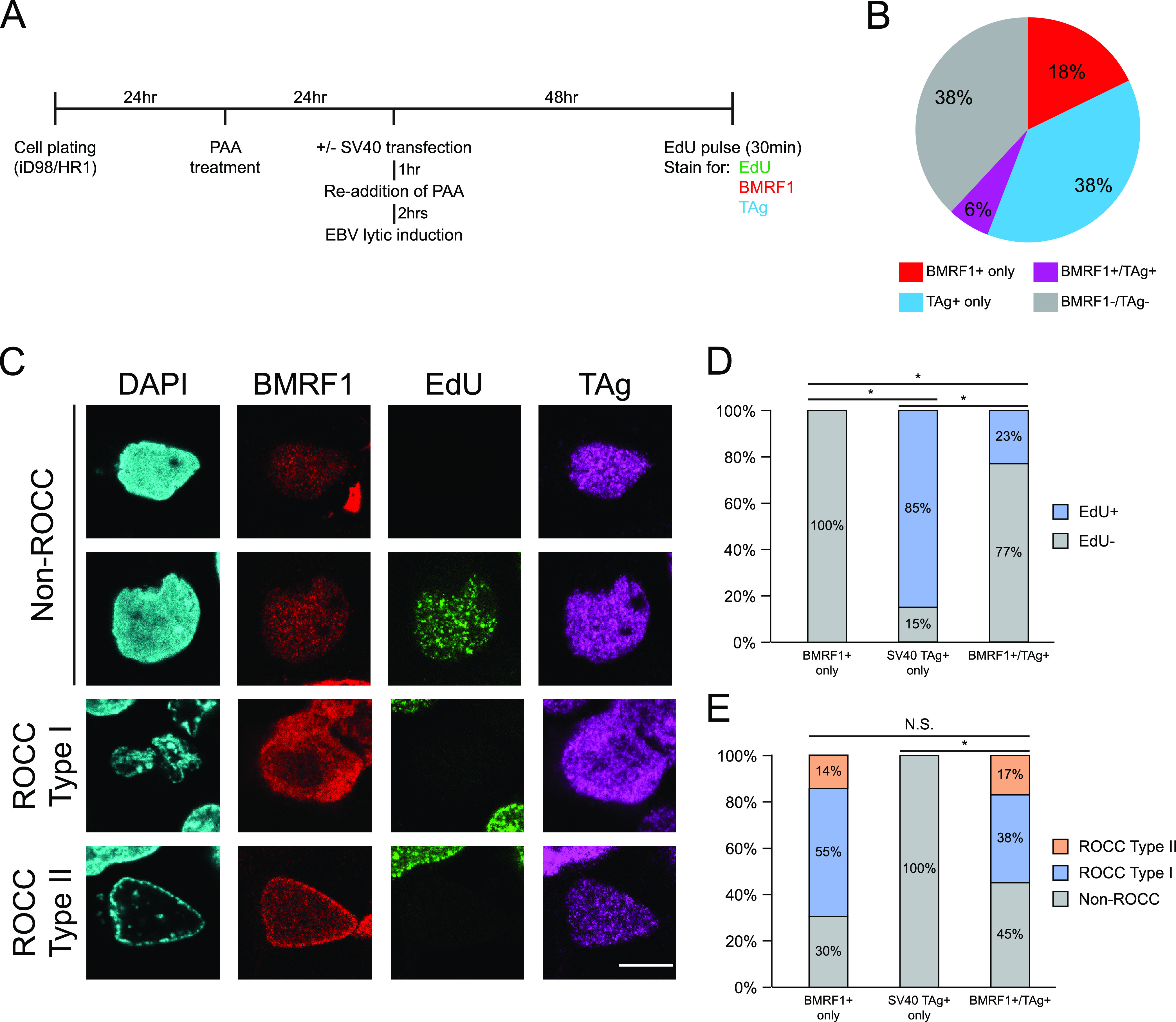
EBV’s ROCC is incompatible with histone-bound DNA replication in lytic cells. (A) Experimental workflow for the EBV and SV40 cotransfection ROCC assay. iD98/HR1 cells were pretreated with 100 μg/mL PAA 24 h prior to transfection. The cells were then mock transfected or transfected with SV40 viral DNA, retreated with PAA, and induced for EBV’s lytic phase by treatment with 200 nM 4-OHT. Forty-eight hours later, cells were pulsed with EdU for 1 h, after which samples were stained for EdU, EBV’s BMRF1, and SV40’s large T antigen (TAg). (B) Proportion of BMRF1^+^, TAg^+^, and BMRF1^+^/TAg^+^ cells in the sample population. *n* = 306 cells. (C) Representative images of samples that were PAA treated, induced for EBV’s lytic phase, and transfected with SV40 viral DNA are shown. All images have the same scale; bar, 10 μm. EdU^+^ signals were found only in BMRF1^+^/TAg^+^ cells displaying non-ROCC. (D and E) EdU status (D) and ROCC types (E) were classified for BMRF1^+^, TAg^+^, or BMRF1^+^/TAg^+^ cells in samples that were PAA treated, induced for EBV’s lytic phase, and transfected with SV40 viral DNA. BMRF1^+^/TAg^+^ double-positive cells have a reduced proportion of EdU^+^ cells compared to TAg^+^-only cells but a similar distribution of ROCC types compared to BMRF1^+^-only cells. This finding indicates that EBV’s ROCC and SV40’s histone-associated DNA amplification are incompatible, and cotransfections favor EBV’s ROCC. *n* > 50 cells per group. For EdU classification (D), *, *P* value of <0.01, Fisher’s exact test. For ROCC classification (E), *, *P* value of <0.01; N.S., *P* value of >0.05, 2 × 3 Fisher’s exact test.

We have tested our model of EBV’s lytic DNA amplification mediating the margination of chromatin, thus driving ROCC type I into ROCC type II, by asking if a different kind of viral DNA amplification, which lacks EBV’s *oriLyt*, could substitute for EBV’s *oriLyt*-mediated DNA amplification in promoting ROCC type II. For this substitution, we chose SV40, a polyomavirus which, like EBV, amplifies and encapsidates its genomes within the nucleus. However, unlike EBV, it encapsidates histone-bound genomes (22) and does not induce ROCC (23). iD98/HR1 cells were plated and treated with 100 μg/mL PAA 24 h prior to mock or SV40 transfection, and then cells were induced to enter EBV’s lytic phase (Fig. 5A). At 48 h posttransfection, cells were incubated for 1 h with EdU. Cells were scored for their expression of EBV’s BMRF1, indicating entry into EBV’s lytic phase, and SV40’s large T antigen, indicating expression from SV40’s viral DNA. The proportions of cells that express BMRF1 alone (BMRF1^+^ only), T antigen alone (TAg^+^ only), or both (BMRF1^+^/TAg^+^) were measured (Fig. 5B). Control experiments showed that, in the presence of PAA, all cells that entered the lytic phase, as noted by their expression of BMRF1, failed to synthesize EBV DNA and were EdU negative (Fig. S7). Moreover, control samples that were pretreated with PAA, mock transfected, and induced to enter EBV’s lytic phase did not incorporate EdU (0% EdU^+^ cells, *n* > 100 cells counted). We therefore used EdU incorporation to identify cells supporting SV40 replication, in those cells that were transfected with SV40 viral DNA.

10.1128/mbio.02686-22.8 FIG S7 PAA-treated lytic cells do not incorporate EdU. iD98/HR1 cells were untreated or pretreated with 100 μg/mL PAA and induced to enter the lytic phase (Fig. 4). Cells were pulsed with EdU for 30 minutes at 24 and 48 h postinduction, following which cells were fixed and permeabilized and underwent click chemistry reaction to detect incorporated EdU as well as immunofluorescence for the early EBV protein BMRF1. For each lytic (BMRF1^+^) cell, ROCC types and EdU signals were classified. In untreated lytic cells, all cells that display ROCC (both type I and type II) were EdU positive. In contrast, all lytic cells that were treated with PAA were EdU negative, indicating successful inhibition of viral DNA synthesis. *n* > 30 cells per group. Download FIG S7, EPS file, 0.6 MB. Copyright © 2023 Rosemarie et al. 2023 Rosemarie et al. https://creativecommons.org/licenses/by/4.0/ This content is distributed under the terms of the Creative Commons Attribution 4.0 International license.

From this experiment, we found that we could not answer our initial question: whether SV40’s DNA amplification could substitute for EBV’s *oriLyt*-mediated DNA amplification in driving ROCC type I into ROCC type II, as it appears that EBV’s ROCC and SV40’s DNA amplification are incompatible (Fig. 5C). Of cells that had entered EBV’s lytic phase and were transfected with SV40 DNA (BMRF1^+^/TAg^+^), only those that had no ROCC were found to support SV40 replication (EdU^+^). In addition, the proportion of total EdU^+^ cells was reduced in the BMRF1^+^/TAg^+^ population compared to cells transfected with SV40 DNA alone (TAg^+^ only) (Fig. 5D). In contrast, the distribution of ROCCs in these BMRF1^+^/TAg^+^ double-positive cells was not significantly different from that of BMRF1^+^ cells (Fig. 5E). Thus, EBV’s ROCC and SV40’s DNA amplification are incompatible, with the cotransfections favoring EBV’s ROCC.

In both the BMRF1^+^-only and BMRF1^+^/TAg^+^ populations of this experiment, there is a higher proportion of cells supporting ROCC type I than those supporting ROCC type II (Fig. 5E), an apparent discrepancy with the population distribution of ROCC types in previous experiments (Fig. 4). This difference can be explained by one condition of the experiments involving transfections of SV40 viral DNA. When EBV-positive cells are induced for the lytic phase, they enter the lytic phase at the beginning of their next S phase (13). Following transfection, cells tend to have a delayed growth/cycle, likely resulting in a later entry into the lytic phase, thus affecting the proportion of ROCC^+^ cells at 48 hpi. Taking this delay into account, the proportions of cells supporting ROCC type I and type II in the experiment involving SV40 transfections are consistent with those previously seen in iD98/HR1 cells induced to enter the lytic phase in the presence of PAA (Fig. 4D).

Our findings from the cotransfection of SV40 viral DNA into EBV lytic cells led us to an additional mechanistic insight: EBV inhibits cellular DNA synthesis during its lytic phase, and we now found that its ROCC is incompatible with SV40 DNA synthesis. Both cellular and SV40 DNA synthesis require cellular DNA polymerases and also are histone associated. Polyomaviruses, such as SV40, as well as papillomaviruses, encapsidate histone-bound genomes and do not induce ROCC (22, 45). In contrast, all five families of DNA viruses that induce ROCC lack cellular histones in their virions (7–10, 46, 47). In EBV, cellular DNA synthesis does not occur once viral DNA synthesis begins. Thus, it is likely that the mechanism that underlies the failure of cellular DNA synthesis extends to that of SV40 through a shared mechanism involving the inhibition of histone-associated DNA synthesis.

## DISCUSSION

Five families of DNA viruses elicit the reorganization of cellular chromatin (ROCC) during their productive infections. However, little was known about the mechanisms by which they mediate ROCC, nor how this compaction of cellular DNA benefits their virus production. We have examined the ROCC driven by EBV to illuminate these unknowns. Our studies with EBV differ from those with other viruses that induce ROCC because EBV usually enters its productive phase having first established its latent phase in newly infected cells.

Given that EBV’s ROCC can first be detected shortly before or coincident with the amplification of its DNA (13), we tested genetically EBV’s DNA synthesis genes for their potential roles in ROCC. Both EBV’s *oriLyt* and its genes encoding the core lytic DNA synthesis complex are required for ROCC (Fig. 1 and Table 1). However, neither BGLF4, which encodes the viral protein kinase, nor EBV’s true late genes contribute to ROCC. These findings yielded the first mechanistic insight into EBV’s eliciting ROCC: the viral productive DNA replication is integral to forming ROCC.

This insight led us to ask if EBV DNA synthesis *per se* is essential for ROCC. We used inhibitors of viral DNA synthesis, ganciclovir (GCV) and phosphonoacetic acid (PAA), and found that an early form of ROCC (type I), in which cellular chromatin became compacted but was not moved to the periphery of the nucleus, took place (Fig. 4). Complete ROCC (type II) required extensive EBV DNA amplification. These surprising findings were extended on examining mutants of EBV’s DNA polymerase encoded by *BALF5.* Catalytically dead derivatives of EBV’s DNA polymerase supported neither type I nor type II of ROCC (Fig. 2). These observations led to a second insight into EBV’s mechanism of mediating ROCC: EBV induces a compaction of cellular chromatin prior to observable amplification of its DNA. It is likely from our findings with the BALF5 mutants and GCV/PAA experiments that this initial compaction requires the formation of a viral replication complex and the initiation of, but not extensive, viral DNA synthesis. Taking these findings together with our results from the transcomplementation assays (Fig. 1), it is apparent that EBV’s *oriLyt* and the core components of the lytic DNA synthesis complex are required for both type I and type II of ROCC, as neither form was observed in the absence of *oriLyt* or each of the core lytic DNA synthesis genes.

Not all families of DNA viruses which replicate their genomes in the nucleus induce ROCC. We asked whether a different kind of viral DNA amplification, which lacks EBV’s *oriLyt*, could substitute for EBV’s *oriLyt*-mediated DNA amplification in promoting ROCC type II. In using SV40, a virus which replicates its DNA in the nucleus but does not induce ROCC (23), we found that EBV’s ROCC and SV40’s DNA replication are incompatible. SV40 can replicate in cells that carry EBV but not ones that have induced ROCC (Fig. 5). It has been shown previously that EBV’s lytic phase inhibits cellular DNA synthesis, and we now found that EBV’s ROCC is incompatible with SV40 DNA replication. A third insight into the mechanism of EBV’s ROCC comes from the observations that both cellular and SV40 DNA synthesis are blocked during EBV’s lytic DNA synthesis. Thus, ROCC occurs when DNA synthesis requiring histone deposition does not occur while EBV’s histone-free DNA synthesis does.

**FIG 6:**
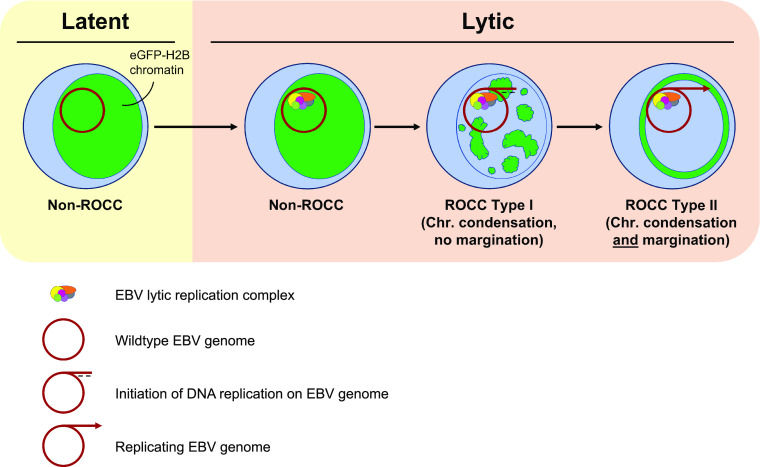
A model for EBV’s reorganization of cellular chromatin (ROCC) during its productive phase. An EBV-infected cell in latency organizes its chromatin (Chr.) indistinguishably from a noninfected cell; that is, cellular chromatin is distributed relatively evenly throughout the nucleus. Upon entry into the viral productive (lytic) phase, EBV’s DNA synthesis complexes form at *oriLyt* sites. In the absence of any one of the DNA synthesis complex components or of *oriLyt*, DNA synthesis initiation fails to take place, and no ROCC occurs. When DNA synthesis complexes do form and initiation takes place, ROCC is induced. ROCC type I, where host chromatin is condensed but not marginated, requires only limited DNA amplification, in which the initiation of viral DNA synthesis triggers a global chromatin condensation. In contrast, ROCC type II, where host chromatin is both condensed and marginated, usually requires extensive amplification of viral DNA.

Our three insights into the mechanisms of ROCC have led us to propose a two-step model (Fig. 6). First, cellular chromatin is triggered to coalesce by the formation of a viral replication complex initiating synthesis in the absence of the deposition of cellular histones. This first step, which we call ROCC type I, is reminiscent of the “honeycombed” nuclear structure previously observed in early lytic EBV cells (13) and the fiber-like structure of chromatin in cells infected with geminiviruses (19). Second, the synthesis of histone-free viral DNA drives the cellular chromatin to the periphery of the nucleus, resulting in its characteristic margination.

EBV’s induction of ROCC may seem counterintuitive to the success of its own lytic DNA replication. While the cellular chromatin is being compacted and marginated, EBV continues to synthesize its DNA. This apparent escape from the global chromatin compaction may be mediated by the viral transactivator protein, Zta. The chromatinized circular EBV genome has many Zta binding sites (48). Zta has been shown to be a pioneer factor, capable of binding “closed” chromatin and keeping it in an “open” conformation (49, 50). It is possible that the binding of multiple Zta proteins along the circular EBV genome contributes to their serving as the templates for lytic DNA synthesis. It has also been demonstrated that newly synthesized, histone-free EBV DNA is capable of serving as the template for DNA synthesis (13). Both events could allow EBV’s lytic DNA synthesis to continue in parallel with ROCC.

Much evidence supports the involvement of cellular factors in the global chromatin compaction that characterizes ROCC type I. The five families of viruses that induce ROCC vary in their genetic content, with geminiviruses and parvoviruses containing as few as two to four genes. Their restricted genetic content means they are unlikely to encode the machinery required directly to condense the cellular chromosomal DNA. For example, some geminiviruses multimerize their capsids to accommodate longer DNAs rather than condensing them (51). Thus, it is likely that viruses promote the chromatin compaction in ROCC by inducing their host to use its own condensation and chromatin remodeling machineries. Cellular factors known to contribute to the remodeling of chromatin structures could contribute to ROCC type I; these include the condensin and cohesin complexes, histone modifications associated with repressed gene expression and heterochromatin, such as H3K9me3, H3K27me3, or macroH2A, and insulator proteins such as CTCF (CCCTC-binding factor).

We have considered two mechanisms to explain how EBV mediates the margination of cellular chromatin. The first depends on EBV’s replication compartments moving the cellular chromatin as they expand. The compartments are thought not to be bound by membranes and do expand and fuse as the productive cycle progresses (32). Although the viral DNA in them can accumulate to 30% of the level of cellular DNA, the accompanying increase in volume of the nucleus means that the concentration of the amplified viral DNA is less than or similar to that of the cellular DNA prior to EBV’s productive phase (32). In this proposed mechanism, the expanding compartment with viral DNA unbound by histones would drive the cellular chromatin by charge repulsion. DNA carries a high negative charge which is about twice that of nucleosome-bound DNA (52). However, the charge dispersion by ions has been found to screen the electrostatic potentials of the DNA helices from electrostatic repulsion beyond a center-to-center distance of ≤30 Å (53), so that electrostatic repulsion alone is unlikely to drive chromatin margination. To illustrate, 10^4^ EBV DNA molecules each separated by 30 Å would occupy a volume of 6 μm^3^, a small fraction of a nuclear volume. A second plausible mechanism is that of liquid-liquid phase separation (LLPS) which has been found likely to contribute to the formation of early replication compartments during HCMV’s productive infection (54). This work found that two early genes of HCMV essential for replication are required for the detected LLPS. The requirement for EBV’s core replication proteins for it to mediate ROCC makes LLPS an attractive mechanism to consider further.

No current model explains all cases of ROCC. We found that 1/10 to 1/4 of the cells in which viral DNA synthesis was inhibited displayed ROCC type II (Fig. 4). These cells had less than 2% of the viral DNA produced in the uninhibited cell population as assayed by qPCR (Fig. 3B and C; compare induced with and without GCV or PAA treatment) and no detectable DNA synthesis as assayed by EdU incorporation (Fig. 4A and B). It is apparent that, in a minority of cases, the condensation of cellular chromatin triggered by formation of EBV’s replication complex can lead to the margination of ROCC type II without substantive viral DNA replication.

How do the many viruses that induce ROCC benefit from this striking reorganization of their host’s chromatin? All these viruses express essential viral genes from chromatin-bound templates to carry out their productive infections. They all package viral DNAs free of cellular histones. ROCC provides them a common pathway to segregate the progeny DNA they will encapsidate from the DNA templates used for transcription. Condensed and inaccessible chromatin is also typically associated with a repression of gene expression. In addition to being condensed, chromatin in ROCC is marginated to the periphery of the nucleus. The nuclear periphery is typically a repressive environment, in which case ROCC may resemble the repressed chromatin of lamina-associated domains (LADs) (55, 56). ROCC could contribute to the suppression of host gene expression to benefit the replication and production of progeny viruses.

ROCC is mediated by multiple families of viruses infecting diverse hosts; it is likely that it has an important role in viral life cycles. Viruses that support ROCC share similarities to our model system, EBV. Other herpesviruses, as well as adenoviruses, parvoviruses, baculoviruses, and geminiviruses, all replicate in the host nucleus, are chromatinized within the host, and package histone-free genomes in their virions. As with EBV, ROCC may confer a benefit on these viruses in segregating histone-rich host chromatin from histone-free regions of viral DNA replication. Compacted and marginated chromatin is also associated with suppressed gene expression, in which case ROCC may play a role in the manipulation of host gene expression at the genomic level. A recent proposed megataxonomy of viruses indicates that the five families of ROCC-inducing viruses do not share a common ancestry (57). Thus, it appears that ROCC has evolved independently in five families of viruses as a shared phenotype that supports productive lytic infection. ROCC also uniquely illustrates viruses’ capacity to control the nuclear structures of their hosts.

## MATERIALS AND METHODS

### Cell lines and culture.

All cell lines were maintained in Dulbecco’s modified Eagle’s medium (DMEM) (Gibco) supplemented with 10% fetal bovine serum (FBS) (HyClone Laboratories) and appropriate antibiotics for selection (described below). All cell culture media were supplemented with 200 U/mL penicillin (MP Biomedicals) and 200 mg/mL streptomycin (MP Biomedicals). All cells were grown at 37°C in a 5% CO_2_ humidified atmosphere. iD98/HR1 is a fusion between D98 cells and the Burkitt lymphoma cell line P3HR1. Both iD98/HR1 and iD98/HR1 eGFP-H2B cells have been previously described (13); inducible Z-ER (EBV’s immediate early protein Zta fused to the estrogen receptor ligand-binding domain) was selected and maintained in these cells with 1 μg/mL puromycin. The following 293 cells infected with mutant EBV BACmids were kind gifts from Ya-Fang Chiu (Chang-Gung University, Taoyuan, Taiwan) and have been previously described (26): 293/EBV ΔBBLF2/3 (MI-405), 293/EBV ΔBBLF4 (MI-80), 293/EBV ΔBSLF1 (MI-317), and 293/EBV ΔBMRF1 (D28). The following 293 cells infected with mutant EBV BACmids were kind gifts from Eric Johannsen (University of Wisconsin—Madison, USA) and have been previously described (27): 293/EBV Δ*oriLyt* and 293/EBV ΔBALF2/HA-BcRF1. The cell lines 293/EBV ΔBALF5, 293/EBV ΔBMLF1, and 293/EBV ΔBGLF4 were obtained from Eric Johannsen. The cell lines 293/EBV ΔBVLF1 and 293/EBV ΔBcRF1 were generated using EBV mutant BACmids obtained from Eric Johannsen and have been previously described (27). EBV-positive 293 cells were selected and maintained with 200 μg/mL hygromycin B.

### Generation of mutant EBV-positive 293 cell lines.

293/EBV ΔBVLF1 and 293/EBV ΔBcRF1 were generated using BM2710 Escherichia coli carrying the invasin gene from Yersinia pseudotuberculosis and the *hly* gene encoding listeriolysin O from Listeria monocytogenes, which allow gene transfer of intact BACmids to some mammalian cells *in vitro*. BACmids EBV ΔBVLF1 (MI-383) and EBV ΔBcRF1 (MI-27) have been previously described as a part of a comprehensive EBV mutant library (26). BM2710 E. coli stably transformed with EBV ΔBVLF1 or EBV ΔBcRF1 was obtained from Eric Johannsen (University of Wisconsin—Madison, USA). These EBV-positive BM2710 E. coli cells were grown overnight at 32°C in brain heart infusion media (37 g/L [wt/vol]) supplemented with 0.5 mM 2,6-diaminopimelic acid (DAP) (Alfa Aesar) and 25 μg/μL spectinomycin (Alfa Aesar) and selected with 50 μg/mL kanamycin. One milliliter of BM2710 E. coli overnight culture was added to 293 cells at 80 to 90% confluence in a 60-mm cell culture plate, in 1× DMEM supplemented with 0.5 mM DAP and 25 μg/μL spectinomycin, and incubated for 2 h. Following incubation, the medium was removed, the cells were washed with 1× DMEM to remove as many bacteria as possible, and fresh 1× DMEM plus 10% FBS medium was added, supplemented with 50 μg/mL gentamicin (Gibco). The following day, the medium was replaced with 1× DMEM plus 10% FBS. EBV-positive 293 cells were selected and maintained with 200 μg/mL hygromycin B.

### Plasmids.

pSG5-BMLF1, pSG5-BMRF1, and pSG5-Rta have been described previously (58) and were obtained from Shannon Kenney (University of Wisconsin—Madison, USA); pCMV5.1/FLAG-BALF5, pCDNA-HA2-BALF2, pCDNA-HA2-BBLF2/3, pCDNA-HA2-BBLF4, and pCDNA-HA2-BSLF1 were obtained from Ya-Fang Chiu (Chang-Gung University, Taoyuan, Taiwan). pCDNA3-HA(2x)-BVLF1 and pCDNA3-HA-BcRF1 were obtained from Eric Johannsen (University of Wisconsin—Madison, USA) and have been previously described (27). Plasmids p509, encoding pCMV-BZLF1 (Zta), and p526, encoding *oriLyt*, have been described previously (59). p3803, a retroviral vector encoding eGFP-H2B, was described previously (13). p3622 is a version of p3803 encoding mCherry-H2B. The wild-type EBV plasmid p2089 has been described previously (60). pCDNA-HA3-BGLF4 was cloned by PCR amplifying *BGLF4* from p2089 using primers 5′-CGCGGGTACCGCCACCATGTATCCATATGACGTTCCAGATTACGCTGATGTGAATATGGCTGCGGAG-3′ (forward) and 5′-CGCGGAATTCTCATCCACGTCGGCCATCTGGACC-3′ (reverse) and cloning it into pCDNA3-HA-SUMO1 plasmid (Addgene plasmid no. 21154) at EcoRI/Acc65I sites.

### Generation of BALF5 mutant expression vectors.

Expression vectors encoding the wild type (Addgene no. 192454) and D755N/D757N (Addgene no. 192455), Y753F (Addgene no. 192456), and S758T (Addgene no. 192457) mutants of EBV’s BALF5 were generated using Gibson assembly. The pCMV5.1/FLAG-BALF5 plasmid, encoding wild-type BALF5 sequence, was digested with NheI-HF (New England Biolabs [NEB], R3131) and FastDigest KflI (isoschizomer of SanDI; Thermo Fisher Scientific; catalog no. FD2164), and the digested DNA was purified using a QIAquick PCR purification kit (Qiagen; catalog no. 28104). Point mutation D755N/D757N, Y753F, or S758T was introduced by Gibson assembly using NEBuilder HiFi DNA assembly master mix (NEB; catalog no. E2621) following the manufacturer’s protocol, with gBlocks gene fragments containing mutated YGDTDS sequences (ordered from Integrated DNA Technologies [IDT]) as inserts at a vector/insert molar ratio of 1:2. Due to GC-level constraints, the gBlocks fragments were designed with several silent nucleotide changes. A gBlocks fragment containing these nucleotide changes but that was wild type in amino acid sequence was used to construct a “codon-shuffled” wild-type BALF5 vector and was used as a control. Resulting Gibson mixes were transformed into NEB 10-beta electrocompetent E. coli (NEB; catalog no. C3020K) and selected by plating onto agar plates containing 100 μg/mL carbenicillin. The integrity of the resulting plasmid clones was verified by Sanger sequencing of the BALF5 regions and Oxford Nanopore sequencing of whole plasmids through Plasmidsaurus (https://www.plasmidsaurus.com/), with results from the latter sequencing deposited onto Addgene as referred to above.

### ROCC transcomplementation assay.

293/EBV single-gene null-mutant cells were plated on 30-mm MatTek plates (MatTek Corporations; catalog no. P35G-1.5-14-C) and transfected under the following conditions: uninduced (eGFP-H2B + carrier), induced (eGFP-H2B + Zta + Rta + carrier), and induced + transcomplemented (eGFP-H2B + Zta + Rta + expression vector for null gene). pCDNA3 was used as a carrier. A total of 5 μg DNA and 5 μL of Lipofectamine 2000 (Invitrogen) were used per plate, and transfection was carried out following the manufacturer’s protocol, with the following modifications: the cells were washed with DMEM, 1 mL DMEM was readded to each plate (half of the typical growth medium volume), and the transfection reagent mixture was gently added to the medium. The transfection mix was incubated with the cells for 4 h in the 37°C cell incubator (humidified, with 5% CO_2_), following which cells were washed with 2 mL of DMEM with 10% FBS (D10F) before another 2 mL of D10F was added as growth medium. Cells were grown for 48 h, and then live-cell imaging was performed using a Zeiss Axiovert 200M fluorescence microscope. ROCC-positive cells were counted and normalized against the number of eGFP-positive cells, and >400 eGFP-positive cells were counted per condition (see Table S1 in the supplemental material) from a total of three transfection replicates. Fisher’s exact test was used for statistical analysis of I versus I+t group pairs of each knockout (KO) cell line. Representative images of cells from transcomplementation assays were taken using the Nikon A1RS HD confocal microscope, to show clearly ROCC from a single, static z-slice.

### Assay for the inhibition of lytic DNA synthesis.

iD98/HR1 eGFP-H2B cells (13) were plated on 22- by 22-mm coverslips in six-well plates and grown overnight. These cells were then treated with either the viral DNA synthesis inhibitor ganciclovir (GCV; 40 μg/mL; Calbiochem) or phosphonoacetic acid (PAA; 100 μg/mL; Sigma), incubated for 24 h, and then induced for EBV’s lytic phase using 200 nM 4-hydroxytamoxifen (4-OHT; Sigma). The concentrations of GCV and PAA used were based experimentally on their inhibiting DNA synthesis optimally, as measured by qPCR and incorporation of EdU, and not being toxic (see also reference 43). At 24 h postinduction (hpi), cells were pulsed with 10 μM 5-ethynyl-2′-deoxyuridine (EdU; Millipore Sigma; catalog no. 900584) for 30 min, at 37°C, and then washed three times with D10F. D10F was added back to the wells along with the appropriate 4-OHT and GCV or PAA concentration. The EdU pulse was repeated at 48 h postinduction, following which cells were fixed for click chemistry and immunofluorescence assays. The effects of GCV and PAA on the viability of cells were measured, and they were found to not affect cell growth in induced populations (data not shown). Additional experiments were performed using a DAPI exclusion assay to measure the viability of cells upon treatment with PAA. Briefly, cells were treated with PAA and induced for EBV’s lytic phase as described above. At 48 hpi, cells were washed twice with 1× Dulbecco’s phosphate-buffered saline (DPBS), stained with 1.5 μg/mL DAPI (Invitrogen) solution, incubated for 10 min at room temperature, and washed twice more with 1× DPBS. Live-cell fluorescence microscopy was used to assess DAPI signals from these cells. Ninety-seven percent of these cells excluded DAPI, indicating that they were viable at 48 hpi, when they were assessed for their ROCC phenotypes (see Fig. S4).

### Quantification of viral DNA copy number by qPCR.

Cells were trypsinized, counted, pelleted, and resuspended in 1× DPBS at a concentration of 10^7^ cells/mL. Cell suspensions were sonicated using a Qsonica Q700 sonicator at 100 mA for at least 1 min. Sonicated cell suspensions were incubated with RNase A (Roche; final concentration, 100 μg/mL) for 30 min at room temperature. Following RNase A treatment, the cell lysates were incubated with proteinase K (Roche; final concentration of 100 μg/mL), 0.1% sodium dodecyl sulfate (SDS), and 1 mM EDTA for 2 to 3 h at 50°C. DNA was isolated from these samples by phenol-chloroform extraction. Briefly, 20 μg of linear acrylamide carrier was added to the samples, they were mixed with an equal volume of 1:1 phenol-chloroform solution and vortexed, and the aqueous phase was again extracted with an equal volume of chloroform. DNA was precipitated from the resulting aqueous solution by ethanol precipitation and resuspended in a small volume of Tris-EDTA (TE) buffer. Fifty nanograms of each purified DNA sample, in triplicate, was subjected to quantitative PCR (qPCR) in a 384-well plate as previously described (61). Briefly, each 20-μL reaction mixture contains 50 ng of the purified sample DNA in a 2-μL volume (diluted in water where necessary), 0.5 μM (each) forward and reverse primers, 0.2 μM probe, 1× ROX reference dye (Invitrogen), and 1× AmpliTaq Gold 360 master mix (Applied Biosystems). The reaction mixtures were incubated at 50°C for 2 min and then at 95°C for 10 min, followed by 40 cycles of 95°C for 15 s and 60°C for 1 min. Measurements were collected on a 7900HT Fast real-time PCR system (Applied Biosciences) and analyzed with the Sequence Detection Systems (SDS) 2.4 from Applied Biosystems. The following primer and probe sequences, specific to EBV *oriP* and cellular rhodopsin, were used: *oriP* (forward, 5′-AGAAGCAGGCGAAGATTCAG-3′; reverse, 5′-CCATTTTAGTCACAAGGGCAG-3′; probe, 5′-/56-FAM/AAGATCAAG/ZEN/GAGCGGGCAGTGAA/3IABkFQ/-3′), rhodopsin (forward, 5′-ATCAGGAACCATTGCCACGTCCTA-3′; reverse, 5′-AGGCCAAAGATGGACACACAGAGT-3′; probe, 5′-/56-FAM/AGCCTCTAG/ZEN/TTTCCAGAAGCTGCACA/3IABkFA/-3′). Standard curves were generated for each primer/probe set at a range of 10^3^ to 10^9^ molecules per well, in triplicate. The following plasmids were used for standard curves: for EBV *oriP*, p152.22 (previously described as pHEBo2 [62]), linearized with BamHI-HF; for rhodopsin, p3934 (a plasmid containing a rhodopsin amplicon), linearized with PvuI. p2134, a plasmid not containing *oriP* or rhodopsin amplicons, was used as carrier. Three technical replicates were used per condition per transfection replicate, with a total of three transfection replicates measured. Statistical analysis was performed using Welch’s two-sample *t* test comparing each condition to the induced (without transcomplementation) condition.

### Click chemistry and immunofluorescence.

Cells grown on 22- by 22-mm coverslips were washed twice with 1× DPBS and fixed with 4% paraformaldehyde (PFA) solution in 1× DPBS (10-min incubation at room temperature). The cells were then washed three times with 1× DPBS, permeabilized with 0.2% Triton X-100 solution in 1× DPBS (10-min incubation at room temperature), and washed three times with 1× DPBS. For EdU detection, coverslips were incubated in freshly prepared click chemistry solution (4 mM CuSO_4_, 50 mM ascorbic acid, 5 μM Cy5-azide [Sigma-Aldrich; catalog no. 777323] in 1× DPBS). Click reaction mixtures were incubated for 30 min, in a dark humidified chamber, at room temperature. Coverslips were then washed three times with 1× DPBS. For immunofluorescence, samples were blocked with a 2% bovine serum albumin (BSA) solution and incubated for 30 min with gentle rocking. Primary antibodies were prepared in in 2% BSA solution, coverslips were incubated with this solution for 1 h at room temperature, and they were washed three times with 1× DPBS. Secondary antibodies were prepared in 2% BSA solution, and coverslips were incubated with this solution for 1 h at room temperature and then were washed three times with 1× DPBS. All antibody incubations were done in a dark, humidified chamber. Coverslips were then carefully dried and mounted with Vectashield (Vector Laboratories) plus 1.5 μg/mL DAPI (Invitrogen). Immunofluorescence images were acquired using a Nikon A1RS HD confocal microscope. Primary antibodies were mouse anti-BMRF1 (EMD Millipore; MAB8186; 1:1,000) and rabbit anti-SV40 large T antigen (GeneTex; GTX134378; 1:250). Secondary antibodies were Alexa Fluor 568 goat anti-mouse (Molecular Probes; A11019; 1:1,000) and Alexa Fluor 647 donkey anti-rabbit (Invitrogen; A31573; 1:500).

### Immunoblotting.

Cells were trypsinized, counted, pelleted, washed with 1× DPBS, repelleted, and resuspended in Lysis buffer (50 mM Tris-Cl [pH 7.4], 1 mM EDTA, 1% Triton X-100, 150 mM NaCl, 1× protease-inhibitor cocktail [PIC; cOmplete, Mini, EDTA-free protease inhibitor cocktail; Roche] in double-distilled water [ddH_2_O]) at a concentration of 10^7^ cells per mL of buffer. The cell suspension was sonicated using a Qsonica Q700 sonicator at 100 mA for at least 1 min and centrifuged at 12,000 × *g* for 10 min, 4°C, and the supernatant was collected for immunoblotting. The collected supernatant was mixed at a 1:1 ratio with 2× SDS sample buffer (0.12 M Tris-Cl [pH 6.8], 2% SDS, 20% glycerol, 0.3 mM bromophenol blue in ddH_2_O). A volume of 45 μL (approximately 4.5 × 10^5^ cells) per sample per well was loaded onto an SDS-PAGE gel (AnyKD Mini-Protean TGX gel; Bio-Rad), the gel was run, and the proteins were transferred to a nitrocellulose membrane (Amersham). The membranes were blocked in Tris-buffered saline (TBS) with 0.1% Tween 20 (TBST) containing 5% milk and incubated with the primary antibody overnight at 4°C. Following treatment with a primary antibody, the membranes were washed with 1× Tris-buffered saline with 0.1% Tween 20 (TBST) and incubated with the secondary antibody for 1 h at room temperature. For BALF5 detection, the membranes were washed again with TBST and incubated with the tertiary antibody for 1 h at room temperature. All antibody dilutions were prepared in 5% milk solution in TBST. Membranes were washed in TBST, developed using a Pierce enhanced chemiluminescence (ECL) Western blotting substrate (Thermo Fisher Scientific) according to the manufacturer’s protocol, and visualized using a ChemiDoc (Bio-Rad). Primary antibodies were rat anti-BALF5 4C12 (a kind gift from Friedrich Grässer, described in reference 63; 1:10), mouse anti-BMRF1 (EMD Millipore; MAB8186; 1:1,000), mouse anti-alpha-tubulin (Sigma-Aldrich; T6199; 1:10,000), and mouse anti-beta-actin (Sigma-Aldrich; A2228; 1:1,000). Secondary antibodies were rabbit anti-rat (Jackson ImmunoResearch; 312-005-003; 20 μg/mL), goat anti-mouse with horseradish peroxidase (HRP) conjugate (Promega; W4021; 1:2,000). Tertiary antibody was goat anti-rabbit with HRP conjugate (Promega; W4011; 1:2,000).

### SV40 cotransfection assay.

iD98/HR1 cells were plated on coverslips in 6-well plates and treated with 100 μg/mL of PAA, 24 h prior to being mock transfected or transfected with isolated SV40 DNA. SV40 DNA isolation was carried out as previously described (64). The transfection mix was incubated with the cells for 4 h in the 37°C cell incubator (humidified, with 5% CO_2_), and then the cells were washed with 2 mL of DMEM with 10% FBS (D10F) before another 2 mL of D10F was added as growth medium. The cells were grown for 1 h, and then 100 μg/mL PAA was added back to the medium. Cells were grown for another 2 h and then were induced to enter EBV’s lytic phase by the addition of 200 nM 4-OHT. At 48 h after transfection, the cells were incubated with 10 μM EdU for 1 h and then fixed for click chemistry and immunofluorescence assays.

### Statistical analysis.

For EBV transcomplementation, GCV/PAA treatment, and SV40 assays, the total number of cells counted per condition exceeded a predetermined number generated using power calculations to obtain a statistical power of 0.9, with α of 0.05, based on preliminary experiments with a smaller sample size. The program Mstat v7.0 was used for power calculations and statistical analyses with Fisher’s exact test (N. Drinkwater, McArdle Laboratory for Cancer Research, School of Medicine and Public Health, University of Wisconsin) and is available for download (https://oncology.wisc.edu/mstat/). R version 4.1.3 was used for statistical analyses using 2 × 3 Fisher’s exact test and Welch’s *t* test and is available for download (https://www.r-project.org/).

## References

[1] Olins DE, Olins AL. 2003. Chromatin history: our view from the bridge. Nat Rev Mol Cell Biol 4:809–814. doi:10.1038/nrm1225. 14570061

[2] Antonin W, Neumann H. 2016. Chromosome condensation and decondensation during mitosis. Curr Opin Cell Biol 40:15–22. doi:10.1016/j.ceb.2016.01.013. 26895139

[3] Huisinga KL, Brower-Toland B, Elgin SCR. 2006. The contradictory definitions of heterochromatin: transcription and silencing. Chromosoma 115:110–122. doi:10.1007/s00412-006-0052-x. 16506022

[4] Babu A, Verma RS. 1987. Chromosome structure: euchromatin and heterochromatin. Int Rev Cytol 108:1–60. doi:10.1016/S0074-7696(08)61435-7. 2822591

[5] Lee JD, IV, Allen MJ, Balhorn R. 1997. Atomic force microscope analysis of chromatin volumes in human sperm with head-shape abnormalities. Biol Reprod 56:42–49. doi:10.1095/biolreprod56.1.42. 9002631

[6] Fuentes-Mascorro G, Serrano H, Rosado A. 2000. Sperm chromatin. Arch Androl 45:215–225. doi:10.1080/01485010050193995. 11111870

[7] Zhao S, He G, Yang Y, Liang C. 2019. Nucleocapsid assembly of baculoviruses. Viruses 11:595. doi:10.3390/v11070595. 31266177 PMC6669607

[8] Wang M, Tuladhar E, Shen S, Wang H, van Oers MM, Vlak JM, Westenberg M. 2010. Specificity of baculovirus P6.9 basic DNA-binding proteins and critical role of the C terminus in virion formation. J Virol 84:8821–8828. doi:10.1128/JVI.00072-10. 20519380 PMC2919018

[9] Tweeten KA, Bulla LA, Consigli RA. 1980. Characterization of an extremely basic protein derived from granulosis virus nucleocapsids. J Virol 33:866–876. doi:10.1128/JVI.33.2.866-876.1980. 16789190 PMC288612

[10] Ostapchuk P, Suomalainen M, Zheng Y, Boucke K, Greber UF, Hearing P. 2017. The adenovirus major core protein VII is dispensable for virion assembly but is essential for lytic infection. PLoS Pathog 13:e1006455. doi:10.1371/journal.ppat.1006455. 28628648 PMC5491326

[11] Monier K, Armas JC, Etteldorf S, Ghazal P, Sullivan KF. 2000. Annexation of the interchromosomal space during viral infection. Nat Cell Biol 2:661–665. doi:10.1038/35023615. 10980708

[12] Strang BL, Boulant S, Chang L, Knipe DM, Kirchhausen T, Coen DM. 2012. Human cytomegalovirus UL44 concentrates at the periphery of replication compartments, the site of viral DNA synthesis. J Virol 86:2089–2095. doi:10.1128/JVI.06720-11. 22156516 PMC3302373

[13] Chiu YF, Sugden AU, Sugden B. 2013. Epstein-Barr viral productive amplification reprograms nuclear architecture, DNA replication, and histone deposition. Cell Host Microbe 14:607–618. doi:10.1016/j.chom.2013.11.009. 24331459 PMC3995538

[14] Besse S, Puvion-Dutilleul F. 1994. Compartmentalization of cellular and viral DNAs in adenovirus type 5 infection as revealed by ultrastructural in situ hybridization. Chromosome Res 2:123–135. doi:10.1007/BF01553491. 8032671

[15] Moulton JE, Frazier LM, Garg SP, Sekhri KK. 1967. Margination of chromatin in dog kidney cells infected with infectious canine hepatitis virus. Am J Vet Res 28:323–333. 4166436

[16] Ihalainen TO, Niskanen EA, Jylhävä J, Paloheimo O, Dross N, Smolander H, Langowski J, Timonen J, Vihinen-Ranta M. 2009. Parvovirus induced alterations in nuclear architecture and dynamics. PLoS One 4:e5948. doi:10.1371/journal.pone.0005948. 19536327 PMC2694274

[17] Nagamine T, Kawasaki Y, Abe A, Matsumoto S. 2008. Nuclear marginalization of host cell chromatin associated with expansion of two discrete virus-induced subnuclear compartments during baculovirus infection. J Virol 82:6409–6418. doi:10.1128/JVI.00490-08. 18434402 PMC2447088

[18] Rushing AE, Sunter G, Gardiner WE, Dute RR, Bisaro DM. 1987. Ultrastructural aspects of tomato golden mosaic virus infection in tobacco. Phytopathology 77:1231–1236. doi:10.1094/Phyto-77-1231.

[19] Bass H, Nagar S, Hanley-Bowdoin L, Robertson D. 2000. Chromosome condensation induced by geminivirus infection of mature plant cells. J Cell Sci 113:1149–1160. doi:10.1242/jcs.113.7.1149. 10704366

[20] Nagar S, Hanley-Bowdoin L, Robertson D. 2002. Host DNA replication is induced by geminivirus infection of differentiated plant cells. Plant Cell 14:2995–3007. doi:10.1105/tpc.005777. 12468723 PMC151198

[21] Chiu Y-F, Sugden B. 2016. Epstein-Barr virus: the path from latent to productive infection. Annu Rev Virol 3:359–372. doi:10.1146/annurev-virology-110615-042358. 27578440

[22] Germond JE, Hirt B, Oudet P, Gross-Bellark M, Chambon P. 1975. Folding of the DNA double helix in chromatin like structures from simian virus 40. Proc Natl Acad Sci USA 72:1843–1847. doi:10.1073/pnas.72.5.1843. 168578 PMC432643

[23] Katzman RB, Seeger M, Rundell K. 2008. SV40 reporter viruses. J Virol Methods 150:7–13. doi:10.1016/j.jviromet.2008.02.013. 18403028 PMC2440643

[24] Fixman ED, Hayward GS, Hayward SD. 1992. *trans*-Acting requirements for replication of Epstein-Barr virus ori-Lyt. J Virol 66:5030–5039. doi:10.1128/JVI.66.8.5030-5039.1992. 1321285 PMC241360

[25] Fixman ED, Hayward GS, Hayward SD. 1995. Replication of Epstein-Barr virus oriLyt: lack of a dedicated virally encoded origin-binding protein and dependence on Zta in cotransfection assays. J Virol 69:2998–3006. doi:10.1128/JVI.69.5.2998-3006.1995. 7707526 PMC188999

[26] Chiu YF, Tung CP, Lee YH, Wang WH, Li C, Hung JY, Wang CY, Kawaguchi Y, Liu ST. 2007. A comprehensive library of mutations of Epstein-Barr virus. J Gen Virol 88:2463–2472. doi:10.1099/vir.0.82881-0. 17698655

[27] Djavadian R, Chiu YF, Johannsen E. 2016. An Epstein-Barr virus-encoded protein complex requires an origin of lytic replication in cis to mediate late gene transcription. PLoS Pathog 12:e1005718. doi:10.1371/journal.ppat.1005718. 27348612 PMC4922670

[28] Lee C-P, Chen J-Y, Wang J-T, Kimura K, Takemoto A, Lu C-C, Chen M-R. 2007. Epstein-Barr virus BGLF4 kinase induces premature chromosome condensation through activation of condensin and topoisomerase II. J Virol 81:5166–5180. doi:10.1128/JVI.00120-07. 17360754 PMC1900198

[29] Chang YH, Lee CP, Su MT, Wang JT, Chen JY, Lin SF, Tsai CH, Hsieh MJ, Takada K, Chen MR. 2012. Epstein-Barr virus BGLF4 kinase retards cellular S-phase progression and induces chromosomal abnormality. PLoS One 7:e39217. doi:10.1371/journal.pone.0039217. 22768064 PMC3387188

[30] Djavadian R, Hayes M, Johannsen E. 2018. CAGE-seq analysis of Epstein-Barr virus lytic gene transcription: 3 kinetic classes from 2 mechanisms. PLoS Pathog 14:e1007114. doi:10.1371/journal.ppat.1007114. 29864140 PMC6005644

[31] Aubry V, Mure F, Mariamé B, Deschamps T, Wyrwicz LS, Manet E, Gruffat H. 2014. Epstein-Barr virus late gene transcription depends on the assembly of a virus-specific preinitiation complex. J Virol 88:12825–12838. doi:10.1128/JVI.02139-14. 25165108 PMC4248913

[32] Nagaraju T, Sugden AU, Sugden B. 2019. Four-dimensional analyses show that replication compartments are clonal factories in which Epstein-Barr viral DNA amplification is coordinated. Proc Natl Acad Sci USA 116:24630–24638. doi:10.1073/pnas.1913992116. 31744871 PMC6900597

[33] Wong SW, Wahl AF, Yuan PM, Arai N, Pearson BE, Arai K, Korn D, Hunkapiller MW, Wang TS. 1988. Human DNA polymerase alpha gene expression is cell proliferation dependent and its primary structure is similar to both prokaryotic and eukaryotic replicative DNA polymerases. EMBO J 7:37–47. doi:10.1002/j.1460-2075.1988.tb02781.x. 3359994 PMC454213

[34] Delarue M, Poch O, Tordo N, Moras D, Argos P. 1990. An attempt to unify the structure of polymerases. Protein Eng 3:461–467. doi:10.1093/protein/3.6.461. 2196557

[35] Hwang CBC, Ruffner KL, Coen DM. 1992. A point mutation within a distinct conserved region of the herpes simplex virus DNA polymerase gene confers drug resistance. J Virol 66:1774–1776. doi:10.1128/JVI.66.3.1774-1776.1992. 1310779 PMC240935

[36] Bernad A, Lazaro JM, Salas M, Blanco L. 1990. The highly conserved amino acid sequence motif Tyr-Gly-Asp-Thr-Asp-Ser in α-like DNA polymerases is required by phage phi 29 DNA polymerase for protein-primed initiation and polymerization. Proc Natl Acad Sci USA 87:4610–4614. doi:10.1073/pnas.87.12.4610. 2191296 PMC54166

[37] Dorsky DI, Crumpacker CS. 1990. Site-specific mutagenesis of a highly conserved region of the herpes simplex virus type 1 DNA polymerase gene. J Virol 64:1394–1397. doi:10.1128/JVI.64.3.1394-1397.1990. 2154619 PMC249264

[38] Liu S, Knafels JD, Chang JS, Waszak GA, Baldwin ET, Deibel MR, Thomsen DR, Homa FL, Wells PA, Tory MC, Poorman RA, Gao H, Qiu X, Seddon AP. 2006. Crystal structure of the herpes simplex virus 1 DNA polymerase. J Biol Chem 281:18193–18200. doi:10.1074/jbc.M602414200. 16638752

[39] Zarrouk K, Piret J, Boivin G. 2017. Herpesvirus DNA polymerases: structures, functions and inhibitors. Virus Res 234:177–192. doi:10.1016/j.virusres.2017.01.019. 28153606

[40] Copeland WC, Wang TSF. 1993. Mutational analysis of the human DNA polymerase α. The most conserved region in α-like DNA polymerases is involved in metal-specific catalysis. J Biol Chem 268:11028–11040. doi:10.1016/S0021-9258(18)82088-9. 8496164

[41] Crumpacker CS. 1996. Ganciclovir. N Engl J Med 335:721–729. doi:10.1056/NEJM199609053351007. 8786764

[42] Matthews T, Boehme R. 1988. Antiviral activity and mechanism of action of ganciclovir. Rev Infect Dis 10:S490–S494. doi:10.1093/clinids/10.Supplement_3.S490. 2847285

[43] Summers WC, Klein G. 1976. Inhibition of Epstein-Barr virus DNA synthesis and late gene expression by phosphonoacetic acid. J Virol 18:151–155. doi:10.1128/JVI.18.1.151-155.1976. 176457 PMC515533

[44] Leinbach SS, Reno JM, Lee LF, Isbell AF, Boezi JA. 1976. Mechanism of phosphonoacetate inhibition of herpesvirus-induced DNA polymerase. Biochemistry 15:426–430. doi:10.1021/bi00647a029. 55273

[45] Favre M, Breitburd F, Croissant O, Orth G. 1977. Chromatin-like structures obtained after alkaline disruption of bovine and human papillomavirus. J Virol 21:1205–1209. doi:10.1128/JVI.21.3.1205-1209.1977. 191643 PMC515661

[46] Gibson W, Roizman B. 1971. Compartmentalization of spermine and spermidine in the herpes simplex virion. Proc Natl Acad Sci USA 68:2818–2821. doi:10.1073/pnas.68.11.2818. 5288261 PMC389533

[47] Johannsen E, Luftig M, Chase MR, Weicksel S, Cahir-McFarland E, Illanes D, Sarracino D, Kieff E. 2004. Proteins of purified Epstein-Barr virus. Proc Natl Acad Sci USA 101:16286–16291. doi:10.1073/pnas.0407320101. 15534216 PMC528973

[48] Ramasubramanyan S, Kanhere A, Osborn K, Flower K, Jenner RG, Sinclair AJ. 2012. Genome-wide analyses of Zta binding to the Epstein-Barr virus genome reveals interactions in both early and late lytic cycles and an epigenetic switch leading to an altered binding profile. J Virol 86:12494–12502. doi:10.1128/JVI.01705-12. 23015699 PMC3497672

[49] Schaeffner M, Mrozek-Gorska P, Buschle A, Woellmer A, Tagawa T, Cernilogar FM, Schotta G, Krietenstein N, Lieleg C, Korber P, Hammerschmidt W. 2019. BZLF1 interacts with chromatin remodelers promoting escape from latent infections with EBV. Life Sci Alliance 2:e201800108. doi:10.26508/lsa.201800108. 30926617 PMC6441497

[50] Buschle A, Mrozek-Gorska P, Cernilogar FM, Ettinger A, Pich D, Krebs S, Mocanu B, Blum H, Schotta G, Straub T, Hammerschmidt W. 2021. Epstein-Barr virus inactivates the transcriptome and disrupts the chromatin architecture of its host cell in the first phase of lytic reactivation. Nucleic Acids Res 49:3217–3241. doi:10.1093/nar/gkab099. 33675667 PMC8034645

[51] Saunders K, Richardson J, Lawson DM, Lomonossoff GP. 2020. Requirements for the packaging of geminivirus circular single-stranded DNA: effect of DNA length and coat protein sequence. Viruses 12:1235. doi:10.3390/v12111235. 33143128 PMC7694086

[52] Gebala M, Johnson SL, Narlikar GJ, Herschlag D. 2019. Ion counting demonstrates a high electrostatic field generated by the nucleosome. Elife 8:e44993. doi:10.7554/eLife.44993. 31184587 PMC6584128

[53] Hamilton I, Gebala M, Herschlag D, Russell R. 2022. Direct measurement of interhelical DNA repulsion and attraction by quantitative cross-linking. J Am Chem Soc 144:1718–1728. doi:10.1021/jacs.1c11122. 35073489 PMC8815069

[54] Caragliano E, Bonazza S, Frascaroli G, Tang J, Soh TK, Grünewald K, Bosse JB, Brune W. 2022. Human cytomegalovirus forms phase-separated compartments at viral genomes to facilitate viral replication. Cell Rep 38:110469. doi:10.1016/j.celrep.2022.110469. 35263605 PMC8924372

[55] van Steensel B, Belmont AS. 2017. Lamina-associated domains: links with chromosome architecture, heterochromatin and gene repression. Cell 169:780–791. doi:10.1016/j.cell.2017.04.022. 28525751 PMC5532494

[56] Briand N, Collas P. 2020. Lamina-associated domains: peripheral matters and internal affairs. Genome Biol 21:85. doi:10.1186/s13059-020-02003-5. 32241294 PMC7114793

[57] Koonin EV, Dolja VV, Krupovic M, Varsani A, Wolf YI, Yutin N, Zerbini FM, Kuhn JH. 2020. Global organization and proposed megataxonomy of the virus world. Microbiol Mol Biol Rev 84:e00061-19. doi:10.1128/MMBR.00061-19. 32132243 PMC7062200

[58] Sarisky RT, Gao Z, Lieberman PM, Fixman ED, Hayward GS, Hayward SD. 1996. A replication function associated with the activation domain of the Epstein-Barr virus Zta transactivator. J Virol 70:8340–8347. doi:10.1128/JVI.70.12.8340-8347.1996. 8970953 PMC190921

[59] Hammerschmidt W, Sugden B. 1988. Identification and characterization of oriLyt, a lytic origin of DNA replication of Epstein-Barr virus. Cell 55:427–433. doi:10.1016/0092-8674(88)90028-1. 2846181

[60] Feederle R, Kost M, Baumann M, Janz A, Drouet E, Hammerschmidt W, Delecluse HJ. 2000. The Epstein-Barr virus lytic program is controlled by the co-operative functions of two transactivators. EMBO J 19:3080–3089. doi:10.1093/emboj/19.12.3080. 10856251 PMC203345

[61] Vereide DT, Sugden B. 2011. Lymphomas differ in their dependence on Epstein-Barr virus. Blood 117:1977–1985. doi:10.1182/blood-2010-05-285791. 21088132 PMC3056644

[62] Sugden B, Marsh K, Yates J. 1985. A vector that replicates as a plasmid and can be efficiently selected in B-lymphoblasts transformed by Epstein-Barr virus. Mol Cell Biol 5:410–413. doi:10.1128/mcb.5.2.410-413.1985. 2983194 PMC366725

[63] Barth S, Pfuhl T, Mamiani A, Ehses C, Roemer K, Kremmer E, Jäker C, Höck J, Meister G, Grässer FA. 2008. Epstein-Barr virus-encoded microRNA miR-BART2 down-regulates the viral DNA polymerase BALF5. Nucleic Acids Res 36:666–675. doi:10.1093/nar/gkm1080. 18073197 PMC2241876

[64] Hertz GZ, Mertz JE. 1986. Bidirectional promoter elements of simian virus 40 are required for efficient replication of the viral DNA. Mol Cell Biol 6:3513–3522. doi:10.1128/mcb.6.10.3513-3522.1986. 3025597 PMC367100

