# Supplementary material for: How Epstein-Barr Virus Induces the Reorganization of Cellular Chromatin
Source: mBio. 2023 Jan 10;14(1):e02686-22. doi: 10.1128/mbio.02686-22 (PMC9973336; doi:10.1128/mbio.02686-22)
Supplement: TEXT S1 [file mbio.02686-22-s0001.docx]

**Supplementary Information 1. Calculation of DNA compaction within EBV virion**

| **EBV**  Capsid diameter: ~100nm  Sphere vol: 5.2 x 10^5^ nm^3^  Genome size: ~165kbp  DNA bp per unit volume: **0.32**  Ratio: **55.45** | **Human cell**  Nucleus diameter: ~10μm  Sphere vol: 520μm^3^ = 520 x 10^9^ nm^3^  Genome size: 3 x 10^9^ bp  DNA bp per unit volume: **5.77 x 10^-3^**  Ratio: **1** |
| --- | --- |

From the ratio of the DNA base pairs per unit volume, we can infer that the DNA within EBV’s capsid is ~55-fold more compacted than the cellular DNA in a typical cell nucleus.

Sphere volume formula: $\frac{\boldsymbol{4}}{\boldsymbol{3}}\boldsymbol{\pi}\boldsymbol{r}^{\boldsymbol{3}}$
