# Supplementary material for: How Epstein-Barr Virus Induces the Reorganization of Cellular Chromatin
Source: mBio. 2023 Jan 10;14(1):e02686-22. doi: 10.1128/mbio.02686-22 (PMC9973336; doi:10.1128/mbio.02686-22)
Supplement: TABLE S1 [file mbio.02686-22-s0003.docx]

**Table S1. Detailed results of measuring ROCC with trans-complementation assays performed on cells that stably maintain mutant EBV genomes with a single gene knocked out.**

|  | **KO Gene Function** | **Uninduced** | | | | **Induced** | | | | **Induced + trans-complemented** | | | |
| --- | --- | --- | --- | --- | --- | --- | --- | --- | --- | --- | --- | --- | --- |
|  |  | **ROCC+** | **ROCC-** | **Total** | **% ROCC+** | **ROCC+** | **ROCC-** | **Total** | **% ROCC+** | **ROCC+** | **ROCC-** | **Total** | **% ROCC+** |
| **WT** | - | 0 | 458 | 458 | 0 | 31 | 605 | 636 | 4.87 | N/A | N/A | N/A | N/A |
| **ΔBALF5*** | DNA Polymerase | 0 | 664 | 664 | 0 | 0 | 481 | 481 | 0.00 | 78 | 481 | 559 | 13.95 |
| **ΔBALF2*** | ssDNA-binding protein | 0 | 635 | 635 | 0 | 1 | 474 | 475 | 0.21 | 19 | 421 | 440 | 4.32 |
| **ΔBBLF2/3*** | Primase-associated factor | 0 | 854 | 854 | 0 | 0 | 806 | 806 | 0.00 | 16 | 826 | 842 | 1.90 |
| **ΔBBLF4*** | Helicase | 0 | 937 | 937 | 0 | 0 | 923 | 923 | 0.00 | 26 | 986 | 1012 | 2.57 |
| **ΔBSLF1*** | Primase | 0 | 890 | 890 | 0 | 1 | 841 | 842 | 0.12 | 67 | 786 | 853 | 7.85 |
| **ΔBMLF1*** | RNA processing and export | 0 | 930 | 930 | 0 | 0 | 901 | 901 | 0.00 | 15 | 883 | 898 | 1.67 |
| **ΔBMRF1*** | Polymerase processivity factor | 0 | 896 | 896 | 0 | 2 | 841 | 843 | 0.24 | 58 | 791 | 849 | 6.83 |
| **ΔoriLyt*** | Origin of lytic replication | 0 | 481 | 481 | 0 | 0 | 438 | 438 | 0.00 | 44 | 462 | 506 | 8.70 |
| **ΔBGLF4** | Serine/Threonine kinase | 0 | 560 | 560 | 0 | 56 | 415 | 471 | 11.89 | 90 | 521 | 611 | 14.73 |
| **ΔBVLF1** | vPIC component | 0 | 666 | 666 | 0 | 25 | 634 | 659 | 3.79 | 14 | 711 | 725 | 1.93 |
| **ΔBcRF1** | vPIC component | 0 | 646 | 646 | 0 | 94 | 568 | 662 | 14.20 | 39 | 697 | 736 | 5.30 |

* p <0.01, Induced (I) vs Induced + trans-complemented (I+t), Fisher’s exact Test
