# Supplementary material for: How Epstein-Barr Virus Induces the Reorganization of Cellular Chromatin
Source: mBio. 2023 Jan 10;14(1):e02686-22. doi: 10.1128/mbio.02686-22 (PMC9973336; doi:10.1128/mbio.02686-22)
Supplement: TABLE S2 [file mbio.02686-22-s0006.docx]

**Table S2. Results of measuring ROCC with 293/EBV ΔBALF5 trans-complementation assay**

|  | **ROCC+** | **ROCC-** | **Total** | **% ROCC+** |
| --- | --- | --- | --- | --- |
| **Induced + trans-complemented (BALF5 WT)** | 116 | 547 | 663 | 17.50 |
| **Uninduced** | 0 | 683 | 683 | 0 |
| **Induced** | 0 | 616 | 616 | 0 |
| **Induced + trans-complemented (BALF5 D755N/D757N)** | 0 | 672 | 672 | 0 |
| **Induced + trans-complemented (BALF5 Y753F)** | 0 | 667 | 667 | 0 |
| **Induced + trans-complemented (BALF5 S758T)** | 0 | 602 | 602 | 0 |
